# Calcitriol and Calcidiol Can Sensitize Melanoma Cells to Low–LET Proton Beam Irradiation

**DOI:** 10.3390/ijms19082236

**Published:** 2018-07-31

**Authors:** Ewa Podgorska, Agnieszka Drzal, Zenon Matuszak, Jan Swakon, Andrzej Slominski, Martyna Elas, Krystyna Urbanska

**Affiliations:** 1 Faculty of Biochemistry, Biophysics and Biotechnology, Jagiellonian University, Gronostajowa 7 Street, Krakow 30-387, Poland; ewa.podgorska@doctoral.uj.edu.pl (E.P.); agnieszka.drzal@doctoral.uj.edu.pl (A.D.); martyna.elas@uj.edu.pl (M.E.); 2 Department of Medical Physics and Biophysics, Faculty of Physics and Applied Computer Science, AGH University of Science and Technology, al. A. Mickiewicza 30, Krakow 30-059, Poland; zenon.matuszak@fis.agh.edu.pl; 3 Polish Academy of Sciences, Institute of Nuclear Physics, Krakow 31-342, Poland; jan.swakon@ifj.edu.pl; 4 Department of Dermatology, Comprehensive Cancer Center Cancer Chemoprevention Program, University of Alabama at Birmingham, Birmingham, AL 35294, USA; aslominski@uabmc.edu

**Keywords:** vitamin D_3_, proton beam radiotherapy, melanoma, in vitro

## Abstract

Proton beam irradiation promises therapeutic utility in the management of uveal melanoma. Calcitriol (1,25(OH)_2_D_3_)—the biologically active metabolite of vitamin D_3_—and its precursor, calcidiol (25(OH)D_3_), exert pleiotropic effects on melanoma cells. The aim of the study was to evaluate the effect of both calcitriol and calcidiol on melanoma cell proliferation and their response to proton beam irradiation. Three melanoma cell lines (human SKMEL-188 and hamster BHM Ma and BHM Ab), pre-treated with 1,25(OH)_2_D_3_ or 25(OH)D_3_ at graded concentrations (0, 10, 100 nM), were irradiated with 0–5 Gy and then cultured in vitro. Growth curves were determined by counting the cell number every 24 h up to 120 h, which was used to calculate surviving fractions. The obtained survival curves were analysed using two standard models: linear-quadratic and multi-target single hit. Calcitriol inhibited human melanoma proliferation at 10 nM, while only calcidiol inhibited proliferation of hamster lines at 10 and 100 nM doses. Treatment with either 1,25(OH)_2_D_3_ or 25(OH)D_3_ radio sensitized melanoma cells to low doses of proton beam radiation. The strength of the effect increased with the concentration of vitamin D_3_. Our data suggest that vitamin D_3_ may be an adjuvant that modifies proton beam efficiency during melanoma therapy.

## 1. Introduction

Because of their high incidence, mortality rates and resistance to the therapy, melanomas are still one the most challenging cancer types for researchers and clinicians [1,2]. Over the last decade, our understanding of the molecular principles regulating melanoma behaviour has improved significantly, leading to new therapies [3,4]. However, even with the new therapeutic approaches the problem of limited efficacy and selective responsiveness of patients still remains [5]. Therefore, new creative approaches and their combinations are required.

Accumulating evidence from a variety of epidemiological and experimental studies confirms in vitro and in vivo anticancer activity of vitamin D_3_ [6,7,8,9]. Those studies indicate that biologically active vitamin D_3_ derivatives may lower the incidence, and inhibit the progression, of various tumours, including melanoma [10,11,12] and sensitize them to radiotherapy [13,14,15,16,17,18]. Moreover, there are reports of an inverse relationship between patient survival and melanoma thickness and 25(OH)D_3_ serum levels, polymorphisms in the genes encoding the vitamin D receptor (VDR), the vitamin D binding protein, expression of the VDR and CYP27B1 expression [19,20,21,22,23,24,25] and complex relations with CYP24A1 [26]. Additionally, it was proposed that VDR plays a role in the development and progression of melanocytic tumours [27].

So far, no studies have been published on a combination of vitamin D_3_ and proton beam irradiation, a therapy with superior dose distribution compared to photon radiation, which is commonly used in the treatment of uveal melanoma. However, reports on the differences in the level of production of free radicals, cell cycle, cell migration inhibition and apoptotic signalling between photon and proton beam radiotherapy [28,29,30,31,32] suggest possible differences in the effect of their combination with vitamin D_3_.

This work aims to examine the influence of calcitriol (1,25(OH)_2_D_3_) and calcidiol (25(OH)D_3_) on the proliferation and response to proton beam radiotherapy of three melanoma cell lines: human SKMEL-188 and hamster BMH Ma and BHM Ab. In the light of existing evidence, we hypothesize that vitamin D_3_ can sensitize melanoma cells to radiation, thus enhancing its effectiveness. Two different models, linear-quadratic and single hit multi-target, were fitted to analyse the surviving curves of the tested melanoma cell lines exposed to combined treatment and to get the most comprehensive picture of proton radiation dose response.

## 2. Results

### 2.1. Impact of Two Metabolites of Vitamin D_3_ on Melanoma Cell Proliferation

**Figure 1:**
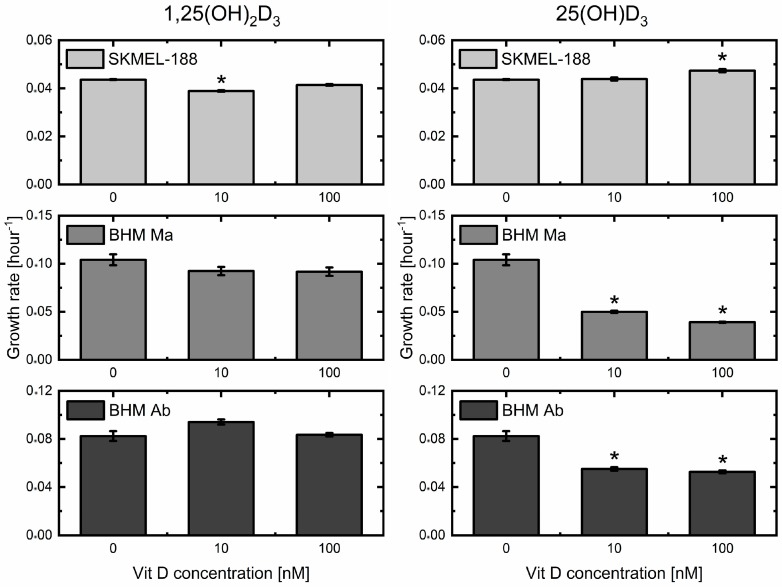
The impact of calcitriol (1,25(OH)_2_D_3_) and calcidiol (25(OH)D_3_) on the proliferation of human SKMel-188 and hamster BHM Ma and BHM Ab melanoma cells. Growth rate was calculated from exponential fitting to cell number, counted every 24 h for 4 days in culture. * denotes statistical significance *p* < 0.05 against control (0 nM of vitamin D_3_ derivative).

1,25(OH)_2_D_3_ and 25(OH)D_3_ affected the growth rate of melanoma cells in culture (Figure 1). In the case of calcitriol, only human SKMEL-188 melanoma cells showed a significant decrease in growth rate, after treatment with the concentration of 10 nM, with BHM Ma and BHM Ab hamster lines showing no major changes in the rate of proliferation. Calcidiol, on the other hand, slightly but significantly stimulated proliferation of SKMEL-188 cells at the concentration of 100 nM, with both BHM Ma and BHM Ab hamster cells showing an inhibition of proliferation at 10 and 100 nM concentrations.

There were also significant differences in basal growth rates between the tested melanoma cell lines. Both hamster lines showed similar proliferation rates (approximately 0.09 h^−1^), while the human SKMEL-188 cells divided almost two times slower. Such differences may be one of the factors influencing response to the vitamin D_3_ metabolites.

### 2.2. Proton Beam Radiosensitivity of Melanoma Cells

**Figure 2:**
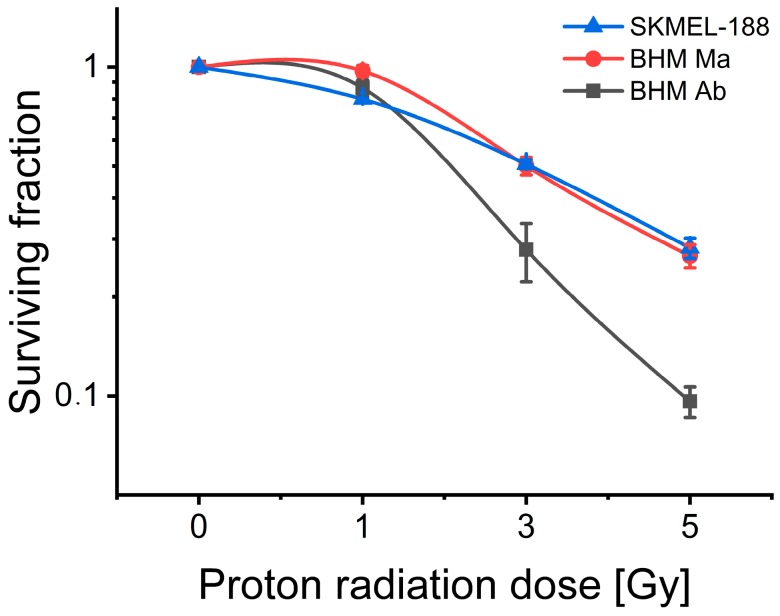
Proton beam radiosensitivity of SKMel-188, BHM Ma and BHM Ab melanoma cells. Surviving fraction was determined from the model described by Buch et al. [34], based on the experimental data from cell number, counted every 24 h for 4 days in culture after irradiation.

Melanoma cell lines are characterized by different radiosensitivity to proton beam therapy (Figure 2), with the highest level of cell killing seen in BHM amelanotic Ab cells. BHM melanotic Ma and SKMEL-188 cells show similar survival curves. Similar differences in response to radiation between BHM Ma and BHM Ab cells were reported for X ray irradiation, where pigmented cells (BHM Ma) were 2.4 times more radio resistant than the unpigmented (BHM Ab) ones [33]. In the case of proton radiation this characteristic is less pronounced but still marked with the ratio of mean lethal dose of 1.56.

### 2.3. Vitamin D_3_ Derivatives Influence Proton Beam Radiosensitivity of Melanoma Cells

**Figure 3:**
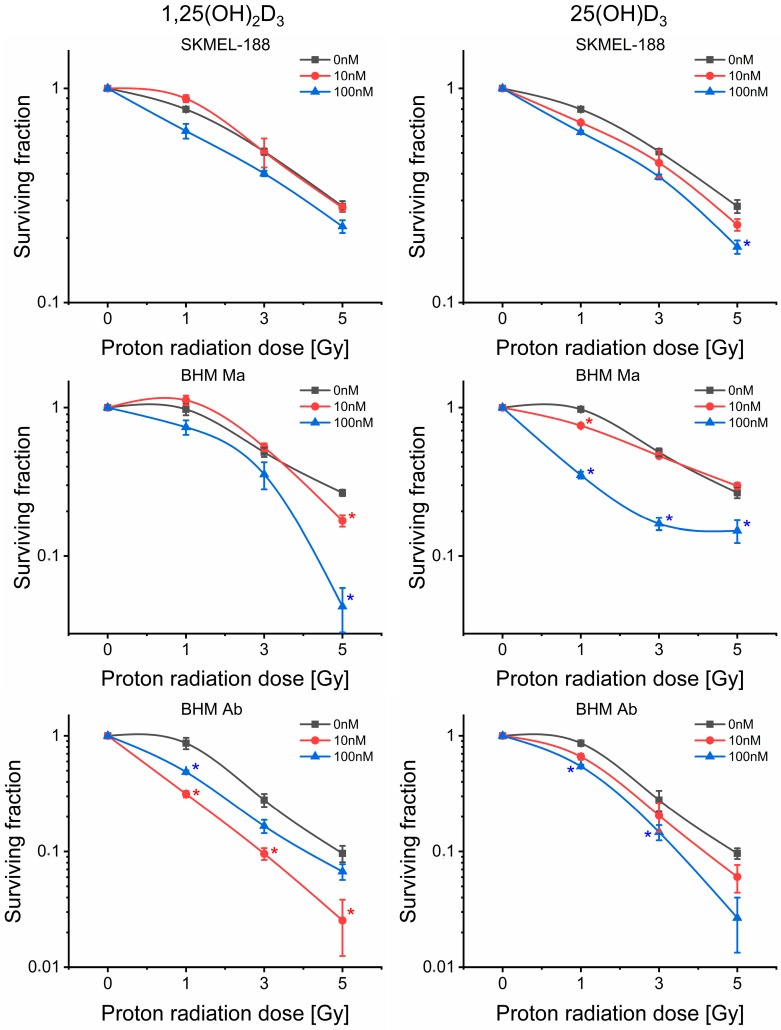
The effect of calcitriol (1,25(OH)_2_D_3_) and calcidiol (25(OH)D_3_) on the cellular response to proton beam irradiation of human SKMel-188 and hamster BHM Ma and BHM Ab melanoma cells. Surviving fraction was determined from the model described by Buch et al. [34], based on the experimental data from cell number, counted every 24 h for 4 days in culture after irradiation. * denotes statistical significance *p* < 0.05 against irradiated control.

**Figure 4:**
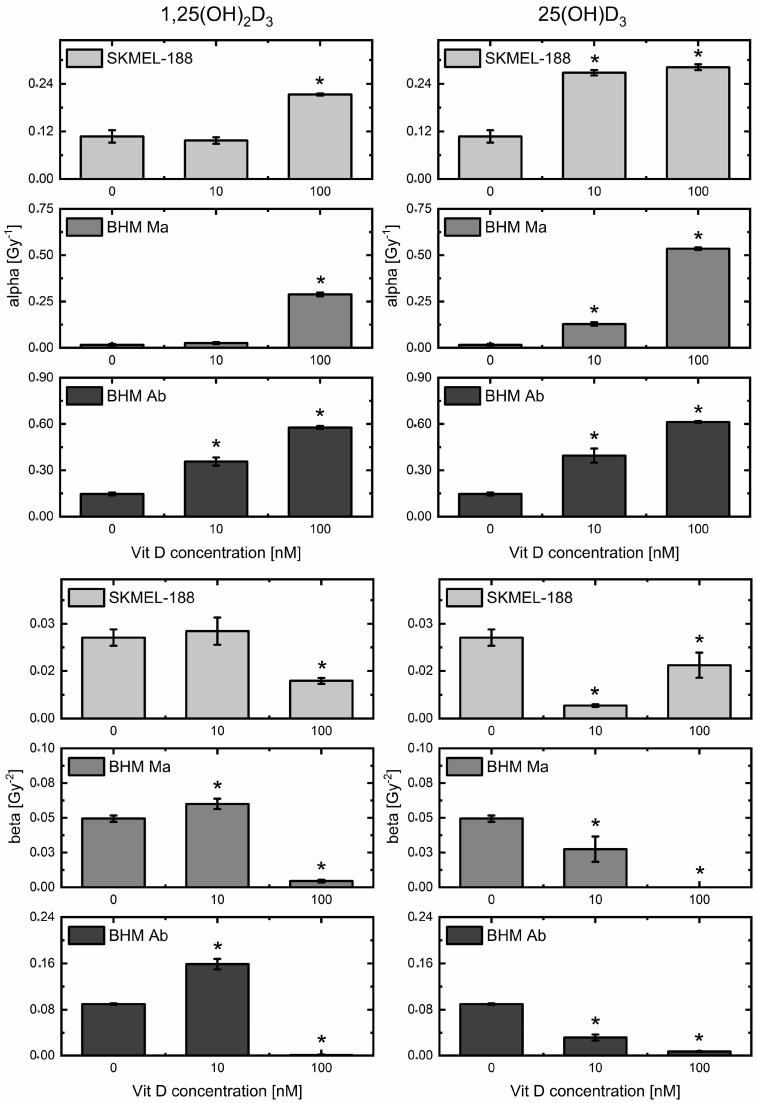
Coefficients α (**upper** panel) and β (**lower** panel) from linear-quadratic model calculated for cells pre-treated with calcitriol (1,25(OH)_2_D_3_) and calcidiol (25(OH)D_3_) and irradiated with proton beam. Surviving fraction was determined from the model described by Buch et al. [34], based on the experimental data from SKMel-188, BHM Ma and BHM Ab cell number, counted every 24 h for 4 days in culture after irradiation. * denotes statistical significance *p* < 0.05 against control (0 nM of vitamin D_3_ derivative).

Changes in both surviving fraction and survival curve shape can be distinguished after pre-treatment with both vitamin D_3_ metabolites (Figure 3). The most significant effects are seen for both vitamin D_3_ derivatives in hamster melanoma lines, with a weaker effect in the human line. Furthermore, higher doses of 25(OH)D_3_ cause in BHM Ma cells a flattening of the survival curve in the region of higher doses (3–5 Gy). Those changes are consistent with the data from fitted survival models. It has been shown that α and β parameters from the linear-quadratic model (LQ model) determine the effectiveness at low and high radiation doses, respectively [35]. Vitamin D_3_ dose dependent decrease in surviving fraction for proton irradiation dose of 1 Gy, visible on the survival curve, is represented by an increase in the values of parameter α (Figure 4, upper panel). For calcitriol, no significant change in α was detected at 10 nM dose for SKMEL-188 and BHM Ma cells. On survival curves, that concentration was slightly protective for cancer cells, with surviving fraction higher than the control one but the effect was not statistically significant. The most effective dose was 100 nM. Calcidiol has a clear dose dependent effect on the radiosensitivity of the studied melanoma cell lines, with the highest concentration being the most effective one. An increase in parameter β, indicating higher effectiveness of high doses of radiation, occurs only for calcitriol pre-treatment: 10 nM dose in BHM Ma and BHM Ab cells (Figure 4, lower panel). The same dose leads to a noticeable decrease in surviving fraction on survival curves. The tested doses were within the range of the normal serum level of calcitriol, which is between 50–120 nM.

**Figure 5:**
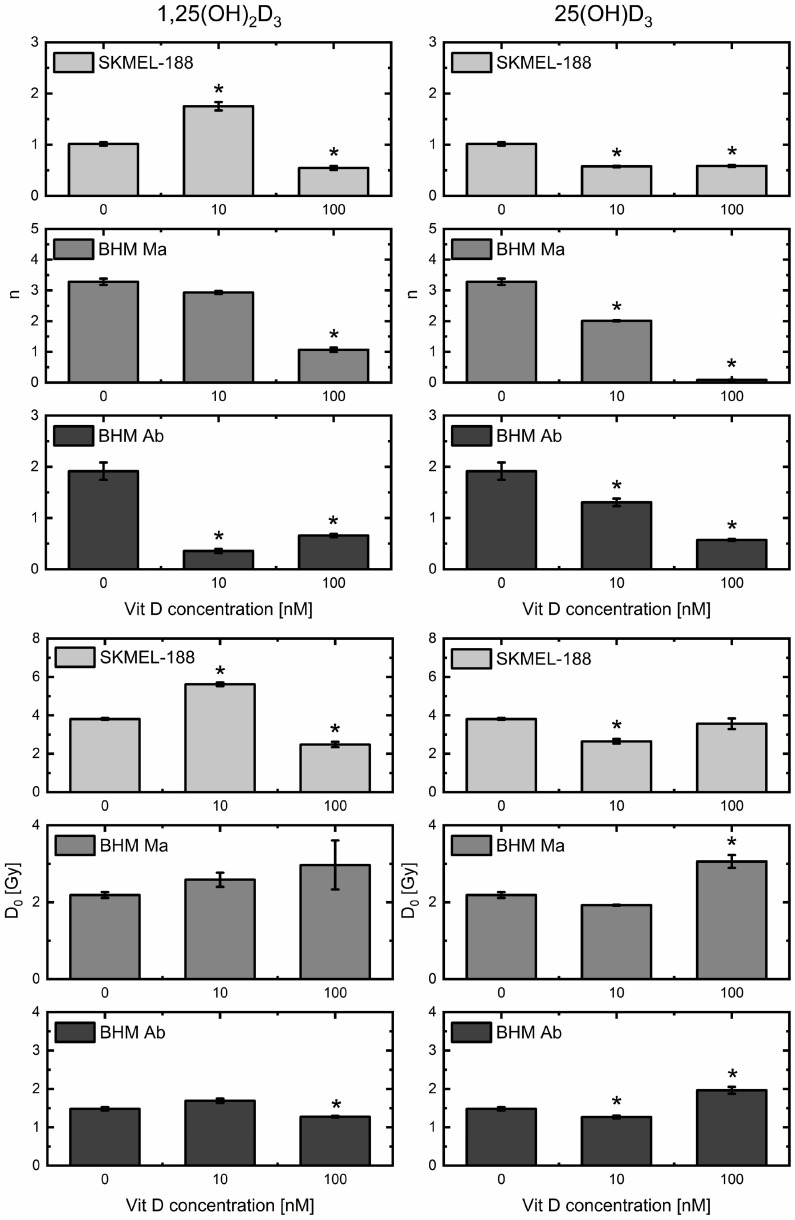
Coefficients *n* (**upper** panel) and D_0_ (**lower** panel) from multi-target single hit model calculated for cells pre-treated with calcitriol (1,25(OH)_2_D_3_) and calcidiol (25(OH)D_3_) and irradiated with proton beam. Surviving fraction was determined from the model described by Buch et al. [34], based on the experimental data from SKMel-188, BHM Ma and BHM Ab cell number, counted every 24 h for 4 days in culture after irradiation. * denotes statistical significance *p* < 0.05 against control (0 nM of vitamin D_3_ derivative).

Results from the second model applied to survival data, single hit multi-target, are in agreement with data from the LQ model. Changes in the calculated parameters *n* and D_0_ are shown in Figure 5. Parameter *n*, indicating the required number of hits for cell death and being sensitive to the effectiveness of low radiation doses, shows an opposite trend to parameter α from the LQ model. An increase in α goes together with a decrease in *n*, which is consistent with vitamin D_3_ metabolites radiosensitizing cells to low doses of proton beam irradiation. The second parameter, mean lethal dose D_0_, describes the average effect of radiation, without distinction between low and high doses [36]. Only several groups were characterized by a decrease in that parameter in comparison to the control, representing the process of radiosensitization by the tested vitamin D_3_ analogues. Among them, there were groups treated with 100 nM of calcitriol, the most effective treatment in enhancing the efficiency of low radiation doses. This indicates that the highest influence of vitamin D_3_ analogues on the averaged effects relates to the response to low doses of proton radiation. 

## 3. Discussion

Current pre-clinical and clinical reports show that vitamin D_3_ has overall anticancer properties [6,7,8]. The finding is also supported by a strong correlation between higher serum 25(OH)D_3_ level and lower incidence of breast [37,38], colon [39], lung [40], prostate [41] and melanoma [10] and by the antiproliferative and pro-differentiative action of vitamin D_3_ and its analogues towards multiple cancer cell lines [42,43,44,45,46,47]. Interestingly, not all cancer cell lines do respond to vitamin D_3_ [20,48,49,50,51], which also included melanoma lines [41,50] and such responsiveness can depend on the culture conditions [11].

In the present study, we demonstrate differential effects of the biologically active form of vitamin D_3_, calcitriol and its precursor calcidiol, on the proliferation rate of three melanoma cell lines: human SKMEL-188 and hamster BHM Ma and BHM Ab (Figure 1). Only SKMEL-188 cells responded to lower doses of 1,25(OH)_2_D_3_ with inhibition of growth rate, which was consistent with other reports [22,52]. However, inhibition of proliferation by calcidiol, with no effect in the case of calcitriol in both BHM Ma and BHM Ab cells requires further explanation. It has been proposed that one of the factors influencing the responsiveness of melanoma cell lines to vitamin D_3_ is melanin pigmentation [53,54], which would explain the decrease in VDR expression with concomitant decrease in anti-proliferative response to 1,25(OH)_2_D_3_ in human melanoma. It is possible that a similar mechanism could underlie the resistance of BHM Ma, which produces melanin pigment also when cultured in a medium with low tyrosine content [55]. However, since BHM Ab cells were pre-selected for amelanotic phenotype by culturing in DMEM, other factors, including a defect in VDR or a high activity of CYP24A1 that inactivates 1,25(OH)_2_D_3_ could be responsible for the differential effects. It must also be noted that there are alternative receptors for vitamin D_3_ derivatives that could affect responsiveness to active forms of vitamin D_3_ (reviewed in [9,10]). Therefore, proper understanding of these differential effects would require additional studies, involving the identification and cloning of hamster nuclear receptors for vitamin D_3_.

Vitamin D_3_ enhances the of anticancer drugs such as doxorubicin, cisplatin, gemcitabine or cyclophosphamide [56,57,58] and sensitizes cancer cells to ionizing radiation [12,13,14,15,16,17,18]. In this paper, we focus on combining calcitriol or calcidiol with proton beam therapy. That mode of cancer therapy is commonly used for the treatment of tumours located near or on critical organs, because of its dosimetric benefits. Thanks to the occurrence of a Bragg peak, there is no exit dose normally deposited by photon therapy within the healthy tissue surrounding the tumour [59]. Accumulating evidence suggests that even with relative biological effectiveness of 1.1, there are significant differences in biological effects on tumour cells between low-energy protons and photon radiation [60]. As regards the combination of proton beam radiotherapy and vitamin D_3_ treatment, the most crucial aspects appear to be the increased production of reactive oxygen species (ROS) and the differences in cell cycle inhibition and apoptotic signalling, all implicated in the response to vitamin D_3_. 

Overall, our results show that vitamin D_3_ is a potential radiosensitizing agent in proton beam irradiation of melanoma cells. Radiosensitization is a complex concept that has many different interpretations and is used to describe a variety of interactions at biochemical and biological level. Alterations in radiosensitization are demonstrated in the SF curve; a downward or leftward shift of the curve implies a radiosensitizing interaction, while an upward or rightward shift implies a radioprotective influence on the treated cells. 

Generally, radiosensitization is conventionally defined as an increased amount of radiation-induced cell death resulting from exposure to a second agent, after correction for the cytotoxicity of the agent (in our case vitamin D_3_, nontoxic in the tested concentrations). Considering the changes in shape and the above-mentioned shift in SF suggests that both calcitriol and calcidiol are radiosensitizing agents, within the range of the concentrations used. Radiosensitization by vitamin D_3_ is particularly visible for low radiation doses, for which we a decrease or elimination of the shoulder on the SF curve is observed (Figure 3). The effect of radiosensitization is also visible at high radiation doses of about 5 Gy. The surviving fraction values of the cells are lower than the analogous values for SF curve obtained for the exposure without vitamin D_3_. Some of the observed survival-dose dependencies, in particular for BHM Ma cells pre-treated with 100 nM of calcidiol, do not resemble the commonly found SF curves with the shoulder in the low dose area and with a linear course for zero for high doses. In this case, we observe a gradual disappearance of the effect the radiation dose has on the number of killed cells. The SF curve gradually flattens (upward-bending) and the number of surviving cells does not decrease with increasing dose. The effect is the reason for the unusual values of some coefficients characterizing survival curves of such non-classical forms of SF, for example, close to zero β values (see Figure 4). The phenomenon (upward-bending of SF) has already been observed during the He-3 irradiation of the V79 cells but the authors interpreted it as an artefact [61]. More recent theoretical studies linked that effect mainly with the repair processes in irradiated cells [62,63]. Nevertheless, the theory is formal and does not explain the upward-bending effect of SF curves with the help of probable molecular mechanisms involved in the repair of irradiated cells. In our work, the upward-bending effect was only observed in the presence of vitamin D_3_ derivatives. Therefore, it might be due to the radioprotective properties of vitamin D_3_. Probable targets of such interaction include ATM and mTor, which are directly related to the process of DNA repair and cell proliferation [64,65,66]. Thus, the radiosensitization effect of high vitamin D_3_ doses would be classified as radiosensitization by targeting the response to DNA damage.

In conclusion, our data suggest that active forms of vitamin D may improve the effectiveness of proton therapy. They also support further in vivo studies on use of vitamin D as adjuvant during radiotherapy of melanoma.

## 4. Materials and Methods

### 4.1. Cell Lines

Human SKMEL-188 melanoma cell line, a gift from Dr Chakraborty, Yale University, was established from a human metastatic melanoma and then maintained in our laboratories as a continuous cell line [67,68].

BHM Ma, Bomirski Hamster Melanoma—pigmented subline, is a stable transplantable tumour cell line that was derived by Dr Andrzej Bomirski in Gdansk, Poland, from a spontaneous hamster melanoma in 1959 [69].

BHM Ab, Bomirski Hamster Melanoma—non-pigmented subline, arose in 1963 by spontaneous alteration of a black tumour (BHM Ma). The amelanotic subline of Bomirski Hamster Melanoma results in non-pigmented tumours in hamsters. The tumours are malignant, dedifferentiated, fast-growing and metastasizing [69]. The cells isolated from those tumours by means of a non-enzymatic method undergo rapid pigmentation in primary cultures in media containing high concentrations of l-tyrosine [70,71]. The level of pigmentation depends on the culture medium; in DMEM with 10% of foetal bovine serum (FBS) the cells are pigmented, while in media low in l-tyrosine, such as Ham’s F10, they are amelanotic [72]. However, culturing for prolonged period in DMEM leads to the selection for an amelanotic phenotype that is stable in cell culture [73]. This type of preselected cells was used in the experiments.

### 4.2. Cell Culture

For each cell line, a different culture medium was used: for BHM Ma—RPMI, for BHM Ab—DMEM and for SKMEL-188 Ham’s F10 medium supplemented with glucose, l-glutamine and pyridoxine hydrochloride. The cells were cultured in 75 cm^2^ flasks in culture medium supplemented with 10% of FBS (Gibco—Thermofisher Scientific, Waltham, MA, USA) and antibiotics (Sigma, St. Luis, MO, USA) and grown until 70% confluent. The calcitriol or calcidiol (Sigma, St. Louis, MO, USA) was dissolved in 100% ethanol to obtain 100 µM stocks. After that stocks were diluted in media to obtain 10 and 100 nM. Concentration of ethanol in media never exceeded 1%. The cells were incubated with vitamin D compounds for 24 h before irradiation. To study the influence of calcitriol or calcidiol on cell proliferation, cell growth curves were determined by manual counting the cells growing with vitamin D_3_ metabolites at different concentrations every 24 h up to 120 h. Counts were performed with the use of haemocytometer, by triplicate by one analyst under a 40× objective according to the standard methodology. Influence on cell response to proton radiation was tested by cultivating cells for 24 h prior to irradiation in a culture medium with 10% FBS, antibiotics and vitamin D_3_ metabolites as indicated. 

### 4.3. Irradiation Procedure

The irradiation was performed at the Institute of Nuclear Physics of the Polish Academy of Sciences (IFJ PAN) in Krakow uses he Proteus C-235 cyclotron produced proton beam. The 230 MeV proton beam, after degradation to 70 MeV, was delivered to the treatment room with a small field horizontal beam line. The passive scattering technique and rotating energy modulator were used to forming the irradiation field. A 40 mm diameter, fully modulated proton beam with energy of 61 MeV, spread-out Bragg Peak (SOBP) with 31.5 mm range and 31.5 mm modulation (measured in water), was used for cell irradiation. Dose of 1, 3, or 5 Gy at dose rates of 1 Gy/min, 2 Gy/min and 6.6 Gy/min respectively, were delivered to the samples. The dose averaged LET_d_ calculated at the depth of 15.8 mm in the SOBP that is, at the centre of the cell container position, was 2.8 keV/µm. The dosimetry was performed according to the recommendations of IAEA TRS-398 protocol [74]. A semiflex ionisation chamber with 0.125 cm^3^ active volume and a PTW reference class UNIDOS Webline electrometer calibrated at the IFJ PAN with ^60^Co radiation source were used. A dedicated PMMA phantom with a holder for the Eppendorf container was used during irradiation. Cells were irradiated in Eppendorf tubes positioned in the phantom, orthogonally to the direction of the proton beam. Cell suspension in phosphate buffer saline (PBS) at 1 × 106 cells/mL was transported on ice between the facilities, including the untreated (non-irradiated) control. Cells were transferred to the culture medium and placed at 1 × 105 cell/mL in 24-well plates. Every 24 h, for 5 days, cells from 6 wells were removed and counted. The experiment was repeated 3 times for each cell line tested.

### 4.4. Data Analysis

Data from cell counting was used to determine cell growth rates, doubling times and surviving fractions. Since the cells in most of the experimental groups exhibited logistic growth, the first step was to establish the duration of exponential growth phase (96 h). Then, growth rates (*gr*):

(1) $$N_{t}=N_{0}e^{gr\cdot t}$$

and doubling times (*t_doubling time_*):

(2) $$t_{doubling\;time}=\frac{\ln\left(2\right)}{gr}$$

where calculated by exponential function fitting, using the nonlinear least squares method. Additionally, time delay for irradiated groups was calculated as previously described [34]. Those values were used to calculate surviving fractions:

(3) $$SF=2^{-\frac{t_{delay}}{t_{doubling\;time}}}$$

Establishing the relationship between the physical conditions of irradiation and its biological effects is a starting point for any radiobiological experiment. Such relations are known as survival curves and presented as the dependence of cell survival probability (Surviving Fractions—SF) on the absorbed radiation dose (in Gy). Many theoretical models of ionizing radiation–induced cell killing have been proposed and described in literature but because the fundamental mechanisms leading to lethal cell damage are not well understood, the models have semi-empirical character and many limitations [36]. Our experimental survival curves were analysed within framework of two classical models describing the response of cells populations to ionizing radiation (IR). The models have a few basic assumptions in common: cell inactivation is treated as a multistep process, cells are killed by energy absorption deposited in a sensitive volume of the cell and radiation-induced lethal events have Poisson distribution. The first one is a “molecular model,” widely known as the linear-quadratic model (LQ):

(4) $$SF\left(D\right)=e^{-\left(\alpha D+\beta D^{2}\right)}$$

describing the logarithmic plot of SF in linear and quadratic dose–dependent terms, where *D* is the absorbed dose. The experimentally determined parameters *α* and *β* are interpreted as rates of cell annihilation by a single-hit and double-hit mechanism, respectively. Molecular interpretation of LQ model is based on the following assumptions: DNA is a critical target, radiation produces the breakage in DNA strands, the broken bonds in DNA strand can be repaired and the critical damage leading to cell death is a double strand break (DSB), resulting in non-repairable lesions. The linear part (*α*) represents a lethal lesion produced by a direct induction of DSB (one track hit) and the quadratic term (*β*) is a result of two single strand breaks (SSB) which could be repaired (a sub-lethal lesion).

The second model used for analysis of our survival curves is a multitarget–single hit (MTSH) model:

(5) $$SF\left(D\right)=1-{\left(1-e^{-\frac{D}{D_{0}}}\right)}^{n}$$

*D*_0_ = 1/k is the dose for 1/e survival in the linear portion of the plot, where k—inactivation constant for each target (or mean lethal dose), *n*—target multiplicity, number of targets per cell that must be inactivated for cell death.

Radiation survival curves are presented as log-linear plots of surviving fraction and dose, respectively; surviving fraction is plotted along the vertical axis (normalized to unity when *D* = 0), radiation dose is plotted along the horizontal axis. 

Both models were fitted to the dose dependence of survival fraction, using the nonlinear least squares method. All analyses were carried out with in-house written Matlab (2014b, MathWorks, Natick, MA, USA) scripts. Optimization of the fitting procedures (both for growth and survival curves) was conducted until mean R-squared from all curves was higher than 0.9. Besides parameters values, their standard errors were calculated and used for the calculation of weighted mean and its error within experimental groups.

### 4.5. Statistical Analysis

Results were presented as weighted mean and weighted mean error. All analyses were performed with STATISTICA 13 software (Stat-Soft Inc., Tulsa, OK, USA), with the use of weights computed during the fitting procedure. For each parameter, Shapiro-Wilk normality test and Levene’s test were used to determine a normal distribution and equality of variances, respectively. Depending on the data, a one-way ANOVA, followed by post-hoc Tukey’s HSD test or Kruskal–Wallis H test, followed by Dunn’s test was performed. *p* values smaller than 0.05 were considered statistically significant. 

## 5. Conclusions

We conclude that the effect of vitamin D_3_ metabolites on the proliferation and response to proton radiation in the studied melanoma cell lines is not straightforward. As regards cell proliferation, the impact of melanin content and other factors such as VDR, CYP27A1, CYP27B1 and CYP24A1 gene expressions as well as VDR localization and interaction with other receptors should be considered in the differential responses of cells to calcitriol and calcidiol. The studied vitamin D_3_ derivatives have proven to be potent radiosensitizers in proton therapy. Radiosensitization by vitamin D_3_ was particularly visible for low radiation doses. Interestingly, for higher doses the observed upward-bending of SF curves in the presence of vitamin D_3_ implies involvement of repair mechanisms and interaction between VDR and radiation-induced signalling pathways. Our results indicate the need for a further investigation of the underlying molecular mechanisms and suggest that vitamin D_3_ can be a promising agent, capable of modifying proton beam therapy efficacy and thus offering a new option in cancer therapy.

## Abbreviations

ATM	Ataxia-telangiectasia mutated kinase
BHM	Bomirski hamster melanoma
CSDA	Continues slowing down approximation
DSB	Double strand break
IR	Ionizing radiation
LQ	Linear-quadratic
mTor	Mammalian target of rapamycin
MTSH	Multitarget—single hit
SF	Surviving fraction
SOBP	Spread-out bragg peak
SSB	Single strand break
VDR	Vitamin D receptor
